# Association between cardiometabolic diseases and the risk and progression of motor neuron diseases in Sweden: a population-based case–control study

**DOI:** 10.1016/j.lanepe.2024.101173

**Published:** 2024-12-10

**Authors:** Charilaos Chourpiliadis, Anikó Lovik, Christina Seitz, Yihan Hu, Jing Wu, Petter Ljungman, Rayomand Press, Kristin Samuelsson, Caroline Ingre, Fang Fang

**Affiliations:** a Institute of Environmental Medicine, Karolinska Institutet, Stockholm, Sweden; b Institute of Psychology, Leiden University, Leiden, the Netherlands; c Department of Cardiology and Clinical Physiology, Danderyd Hospital, Stockholm, Sweden; d Department of Clinical Neuroscience, Karolinska Institutet, Stockholm, Sweden; e Department of Neurology, Karolinska University Hospital, Stockholm, Sweden

**Keywords:** Amyotrophic lateral sclerosis, Cardiometabolic diseases, Cardiac arrhythmia, Heart failure, Thromboembolic disease, Hypertension, Cerebrovascular disease, Ischemic heart disease, Diabetes mellitus type 2, Hypercholesterolemia

## Abstract

**Background:**

The evidence on the link between cardiometabolic diseases (CMDs) and motor neuron diseases (MNDs) remains inconsistent. We aimed to determine whether there is an association of CMDs, namely, any cardiovascular disease, cardiac arrhythmia, heart failure, thromboembolic disease, hypertension, cerebrovascular disease, ischemic heart disease, diabetes mellitus type 2, and hypercholesterolemia with the risk and progression of MNDs.

**Methods:**

We included 1463 MND patients (amyotrophic lateral sclerosis (ALS), primary lateral sclerosis (PLS), progressive spinal muscular atrophy (PSMA), and unspecified MND) diagnosed from January 1, 2015, to July 1, 2023, in Sweden according to the Swedish Motor Neuron Disease Quality Registry (i.e., cases), up to 5 MND-free population controls per case (N = 7311) who were individually matched to the cases on age and sex, and the full siblings (N = 2002) and spouses (N = 1220) of MND patients (i.e., relative controls). Conditional logistic regression models were used to estimate the risk of MND diagnosis in relation to previous CMDs, through comparing MND patients to population controls or relative controls. MND patients were followed from diagnosis to assess the role of pre-diagnostic CMDs on disease progression. A joint longitudinal-survival model was used to estimate risk of mortality (or use of invasive ventilation) in relation to CMDs after taking into account the longitudinal changes of ALS functional rating scale-revised (ALSFRS-R) in the time-to-event analysis. Hierarchical clustering with the Ward's linkage and a dissimilarity matrix created by Gower's method was used to identify clusters of MND patients with distinct phenotypes.

**Findings:**

Among the CMDs studied, a history of diabetes mellitus type 2 (OR 0.75; 95% CI 0.62, 0.93) or hypercholesterolemia (OR 0.82; 95% CI 0.71, 0.94) more than one year before diagnosis was associated with a lower risk for MNDs. The associations persisted for more than five years before MND diagnosis. MND patients with a history of any cardiovascular disease (HR 1.43; 95% CI 1.13, 1.81), arrhythmia (HR 1.42; 95% CI 1.04, 1.93), heart failure (HR 1.79; 95% CI 1.02, 3.14), hypertension (HR 1.41; 95% CI 1.12, 1.77), or hypercholesterolemia (HR 1.28; 95% CI 1.01, 1.62) had an increased mortality risk, compared to others, after taking into consideration the longitudinal changes in ALSFRS-R. Cluster analysis identified two clusters of MND patients, where one cluster demonstrated higher age, worse functional status, and higher prevalence of CMDs at the time of diagnosis as well as a higher mortality and faster functional decline during follow-up, compared to the ones included in the other cluster.

**Interpretation:**

Diabetes mellitus type 2 and hypercholesterolemia were associated with a lower future risk of MND. On the other hand, most of the CMDs were indicative of a poor disease progression after an MND diagnosis.

**Funding:**

10.13039/501100000781European Research Council, US Center for Disease Control and Prevention, 10.13039/501100004359Swedish Research Council.

**Research in context**

**Evidence before this study**

We searched all articles in PubMed using combinations for the following search terms: “Motor neuron disease”, “Arrhythmia”, “Heart failure”, “Thromboembolic disease”, “Hypertension”, “Cerebrovascular disease”, “Ischemic heart disease”, “Diabetes mellitus type 2”, and “Hypercholesterolemia” with their synonyms, from inception up to September 25, 2024, without any language restrictions. Studies analyzing cardiovascular diseases that developed after the onset of MND symptoms were excluded. We included 28 studies exploring the link between cardiometabolic diseases (CMDs) and the risk or progression of motor neuron disease (MND), though the evidence was often contradicting. Few studies have examined these associations across different time windows from decades before until years after MND diagnosis or accounted for familial confounding. Few studies explored the impact of CMDs on survival of MND patients, while none of them considered the effect of informative censoring in their estimates. Finally, none addressed the link between CMDs and MND disease progression.

**Added value of this study**

To our knowledge, this is the first study that examined comprehensively the role of CMDs not only on the risk, but also the prognosis of MND, using a population-based design, with a large sample size, complete follow-up, and rich data on clinical characteristics and sociodemographic factors of the study population. We also studied temporal associations between previous CMDs and the risk of MND diagnosis. Finally, we identified clusters of MND patients, incorporating information on clinical characteristics and pre-diagnostic CMDs, which are indicative of patient outcomes (i.e., survival and functional decline after diagnosis).

**Implications of all the available evidence**

Our findings highlight the importance of CMDs for the prognosis of MND. Monitoring closely the cardiometabolic health of MND patients might be beneficial for the prognosis of this population. Future epidemiological studies should consider time windows when studying potential risk factors for MND to differentiate between upstream and downstream events relative to MND onset.

## Introduction

Motor neuron diseases (MND) are a group of neurodegenerative disorders causing deterioration of voluntary muscle movement.^1^ Amyotrophic lateral sclerosis (ALS) is the most common of these diseases. The existing treatment options for ALS extend life by only a couple of months on average, despite extensive efforts in research.^2^ Identification of risk factors for ALS is therefore crucial for increasing the understanding of the disease and for the development of preventive measures and therapeutic strategies.

Cardiometabolic diseases (CMDs) are prevalent in the population, but, in most people, can be prevented with lifestyle changes or controlled with the required medication.^3^ Emerging evidence suggests a potential link between CMDs and ALS.^4^ Previous research has found that history of diabetes mellitus type 2 might have a protective effect on the risk of ALS,^5^, ^6^, ^7^ although one study found no association.^8^ The evidence regarding hyperlipidemia and risk of ALS is inconsistent, with one study indicating no association,^9^ while others suggesting either a negative^10^, ^11^, ^12^ or positive^13^, ^14^, ^15^ association. Similarly, conflicting results exist between a history of cardiovascular disease (CVD) and risk of ALS.^16^, ^17^, ^18^ While findings on the link between CMDs and risk of ALS remain inconsistent, there is relatively little known regarding the link between CMDs and prognosis of ALS. Among CVDs, only hypertension and coronary heart diseases have been previously examined in relation to ALS survival and only two studies have to date explored coronary heart disease, cardiac arrhythmia, and heart failure in relation to disease progression of ALS.^4^ Diabetes mellitus has been previously studied in relation to ALS survival without clear results^19^, ^20^, ^21^, ^22^ and, similarly, the results from studies on hyperlipidemia and ALS survival are also inconsistent.^9^^,^^13^^,^^23^, ^24^, ^25^, ^26^ Potential explanations for the conflicting findings in the existing literature might include insufficient statistical power in relation to small sample size, selection bias due to the suboptimal representativeness of study sample in relation to its source population, and potential reverse causation (i.e., inability to separate upstream events from downstream events of ALS). Furthermore, to the best of our knowledge, CMDs have not been previously studied with other non-ALS MNDs.

In this study, we first aimed to explore the associations of CMDs, namely any CVD, cardiac arrhythmia, heart failure, thromboembolic disease, hypertension, cerebrovascular disease, ischemic heart disease, diabetes mellitus type 2, and hypercholesterolemia, with the risk of MNDs, using a population-based case–control study with multiple control groups. Secondly, we examined the association of pre-diagnostic CMDs with the risk of death and functional decline after MND diagnosis. Finally, we aimed to cluster MND patients with distinct phenotypical characteristics, through incorporating information on pre-diagnostic CMDs.

## Methods

### Study population

The Swedish Motor Neuron Disease (MND) Quality Registry is a nationwide registry established in 2015.^27^ The Registry collects information on clinical characteristics, biological measurements, and quality of life outcomes from around 80% of MND patients in Sweden and all MND patients in Stockholm.^27^

In the present study, we included 1463 patients with ALS (definite, probable or possible ALS according to the revised El Escorial criteria^28^; n = 1057), primary lateral sclerosis (PLS; n = 40), progressive spinal muscular atrophy (PSMA; n = 61), and unspecified MND (n = 305) diagnosed from January 2015 to July 2023 through the MND Quality Registry and collectively named them as patients with MNDs. To ensure diagnostic consistency, all patients were assessed using the revised El Escorial criteria, in addition to the Gold Coast criteria. All MND patients underwent a standardized diagnostic workup, including magnetic resonance imaging (MRI), neurophysiology exams, lumbar puncture, and genetic screening. A neurologist with a subspecialty in MNDs evaluated each patient to confirm the diagnosis. The patients with a diagnosis of unspecified MND were patients referred to the neurology clinic with suspicion of MND, including progressive weakness symptoms, for whom findings from one or more of the investigations in the diagnostic workup were inconclusive.

We followed all patients from date of diagnosis until date of death, initiation of invasive ventilation, emigration from Sweden, or end of follow-up (September 2023), whichever came first. For each patient, five MND-free population controls of the same age and sex were randomly selected from the Swedish Total Population Register, using the incidence density sampling method.^29^ We selected five controls per case, as a ratio of four to five controls per case strikes a balance between cost-effectiveness and statistical power.^30^^,^^31^ In addition, we identified all the MND-free full siblings (n = 2002) and both past and current spouses (n = 1220) of the MND patients as relative controls, through Statistics Sweden. The date of MND diagnosis was used as the index date for the patient and their population as well as relative controls. Supplementary Figure S1 shows a detailed description of the study design.

### Exposure ascertainment

Information on CMDs before index date was identified for both the MND patients and their respective controls, through linkage with the Swedish Patient Register, which includes information on inpatient care since 1964 (nationwide since 1987) and on specialized outpatient care since 2001.^32^ The validity of the Swedish Patient Register is generally high, with a positive predictive value of around 85–95% for most of the inpatient diagnoses.^32^ We used the 9th (until 1996) and 10th (since 1997) Swedish revisions of the International Classification of Disease (ICD) codes to identify diagnosis of CMDs between January 1st, 1987 and the index date, for both the cases and controls. We considered both primary and secondary diagnoses in relation to a hospital visit. We further linked the cases and controls to the Swedish Prescribed Drug Register, which has since July 2005 included information on prescribed medications in all Swedish pharmacies, to identify CMDs not attended by specialist care (thereby not included in the Patient Register), using Anatomical Therapeutic Chemical (ATC) codes. All ICD and ATC codes used to identify CMDs are shown in Supplementary Table S1.

### Covariables

We ascertained information on date of birth and sex through the Total Population Register. Information on socioeconomic status, country of birth, and educational attainment was obtained from the Swedish Censuses in 1965–1990 and “The longitudinal integrated database for health insurance and labor market studies” (LISA) from 1990 onward.

Detailed clinical information for the MND patients at the time of diagnosis and during follow-up (every 6 months) was retrieved through the MND Quality Registry, including site of disease onset, diagnostic delay, disease progression rate at diagnosis, ALSFRS-R scores, familial ALS, body mass index (BMI), and use of gastrostomy and invasive ventilation.^33^

### Statistical analysis

#### Risk of MND diagnosis

We compared MND patients with population controls using conditional logistic regression to estimate odds ratio (OR) of MND with 95% confidence interval (95% CI) in relation to a previous diagnosis of CMDs. We first examined the associations for CMDs diagnosed within 1 year, 1–5 years, or >5 years before the index date. In the following analyses, we excluded CMDs diagnosed within 1 year before the index date, as the median diagnostic delay of MND was 12.4 months in the study population. All analyses were inherently controlled for age and sex (used as stratum indicator in conditional logistic regression), as the cases and the controls were individually matched on these variables. In all analyses we additionally controlled for socioeconomic status, educational attainment, and country of birth. Second, we compared MND patients with their sibling and spouse controls in terms of history of CMDs and contrasted the results of these comparisons to the ones obtained from the population comparison, to understand the influence of potential familial confounding due to shared genetic and non-genetic factors between siblings or spouses. In the latter analyses, we controlled for age, sex, socioeconomic status, educational attainment, and country of birth, in addition to using family identification number as the strata in the conditional logistic regression.

#### Progression of disease

We estimated the risk of death after an MND diagnosis in relation to pre-diagnostic CMDs (i.e., any time before diagnosis) as a hazard ratio (HR) with 95% confidence interval (95% CI) using a joint longitudinal-survival model, taking into account the time-varying ALFRS-R scores. The joint model combines a longitudinal component with random intercept, slope, and unstructured covariance matrix and a Weibull survival model. The longitudinal and survival components were linked through the shared random effects. Attained age was used as the underlying time scale, and the date of birth as the time origin. The model was adjusted for age at diagnosis, sex, BMI at diagnosis, diagnostic delay, progression rate at diagnosis, and site of onset. The proportionality assumption was tested using Schoenfeld residuals, and interactions with time were introduced for predictors violating the assumption. To assess the soundness of results to the use of time scale, we also performed the survival analysis using time since onset, instead of attained age, as the time scale in a sensitivity analysis.

As a sensitivity analysis to assess the robustness of the joint model, we first performed the same analysis using Cox model, after adjustment for age at diagnosis, sex, onset site, diagnostic delay, BMI at diagnosis, and progression rate at diagnosis. Then, we used a linear mixed model with random intercept and slope to study the average change in ALSFRS-R score over time (estimated as the β coefficient with 95% CI) and compared such among MND patients with and without a history of CMDs. Time was measured from the first ALSFRS-R measurement in intervals of every 6 months. The robust sandwich estimator was included in the model to calculate standard errors. The model was adjusted for age at diagnosis, sex, onset site, diagnostic delay, and BMI at diagnosis. Furthermore, we excluded individuals with a diagnosis of non-ALS MND to examine whether our findings were identical among patients with ALS.

Finally, hierarchical clustering using the Ward's linkage with a dissimilarity matrix based on Gower's method was used to find clusters of MND patients with distinct phenotypes. This descriptive clustering analysis grouped MND patients based on history of the studied CMDs and predictors of disease progression, namely, sex, age at diagnosis, ALSFRS-R at diagnosis, progression rate at diagnosis, diagnostic delay, onset site, and BMI at diagnosis. We determined the number of clusters by assessing associations for survival using joint longitudinal-survival models (estimated as a hazard ratio (HR) with 95% confidence interval (95% CI)) and for the rate of decline in ALSFRS-R score after MND diagnosis using linear mixed models (estimated as the β coefficient with 95% CI), selecting the model with maximized fit and minimized complexity. We then examined the risk of death or the average decline of ALFRS-R score (every six months) comparing MND patients of different clusters. In all analyses we included MND patients with complete information on all predictors because of the relatively large number of patients with missing data in some of the variables (i.e., ALSFRS-R and site of onset) (Supplementary Table S2).

Statistical analyses, including the cluster analysis, were performed using STATA (*Release 16*. College Station, TX: Stata Corp LLC.). Data reporting followed the STROBE (STrengthening the Reporting of OBservational studies in Epidemiology) guidelines (Supplementary File S1).

### Standard protocol approval, registration, and patient consent

The study was approved by the Swedish Ethical Review Authority (2022-02314-01).

### Role of the funding source

The funders had no role in study design, data collection, data analysis, interpretation, or writing of the report.

## Results

In the population comparison, the mean (standard deviation (SD)) age at index date was 67.3 (11.7) years, and 55.6% were men (Table 1). MND patients had a mean (SD) BMI of 23.9 (4.2) kg/m^2^ and ALSFRS-R score of 36.4 (8.2) at the time of diagnosis. Spinal onset was observed in 468 (32.0%) of MND patients. The median (interquartile range, (IQR)) diagnostic delay was 12.4 (7.6–19.8) months.

**Table 1:** Baseline characteristics of patients with MND, their MND-free siblings and spouses as well as age- and sex-matched population controls.

Characteristics	Comparison with population controls		Comparison with sibling controls		Comparison with spouse controls	
	Cases	Population controls	Cases	Siblings	Cases	Spouses
**Number of participants, N**	1463	7311	993	2002	1135	1220
**Age at the index date, mean (SD) [N]**	67.3 (11.7) [1463]	67.3 (11.7) [7311]	66.3 (11.3) [993]	66.4 (12.2) [2002]	67.9 (11.3) [1135]	66.9 (12.4) [1220]
**Male, N (%)**	814 (55.6%)	4067 (55.6%)	567 (57.1%)	981 (49.0%)	640 (56.4%)	520 (42.6%)
**Educational attainment, N (%)**						
<9 years	152 (10.4%)	979 (13.4%)	77 (7.7%)	218 (10.9%)	115 (10.1%)	107 (8.8%)
9–10 years	147 (10.0%)	792 (10.8%)	99 (9.9%)	203 (10.1%)	109 (9.6%)	109 (9%)
Upper secondary education	609 (41.6%)	3180 (43.5%)	425 (42.9%)	849 (42.5%)	473 (41.7%)	519 (42.5%)
Post-Secondary <2 years	82 (5.6%)	356 (4.9%)	66 (6.6%)	103 (5.1%)	58 (5.1%)	47 (3.8%)
Post-Secondary ≥2 years	439 (30.0%)	1849 (25.3%)	310 (31.2%)	524 (26.1%)	349 (30.7%)	395 (32.4%)
Postgraduate education	26 (1.8%)	80 (1.1%)	14 (1.4%)	31 (1.5%)	24 (2.1%)	36 (2.9%)
Missing	8 (0.6%)	75 (1.0%)	2 (0.2%)	74 (3.7%)	7 (0.6%)	7 (0.6%)
**SES, N (%)**						
Occupation without educational requirements	62 (4.2%)	443 (6.1%)	37 (3.7%)	94 (4.7%)	42 (3.7%)	39 (3.2%)
Occupation requiring high school degree	634 (43.3%)	3504 (47.9%)	434 (43.8%)	846 (42.3%)	489 (43.1%)	492 (40.3%)
Occupation requiring university studies ≤3 years	265 (18.1%)	1069 (14.6%)	198 (19.9%)	299 (15%)	217 (19.1%)	201 (16.5%)
Occupation requiring university studies >3 years	361 (24.7%)	1444 (19.8%)	261 (26.2%)	438 (21.9%)	292 (25.7%)	345 (28.3%)
Missing	141 (9.6%)	851 (11.6%)	63 (6.3%)	325 (16.2%)	95 (8.4%)	143 (11.7%)
**Country of birth, N (%)**						
Sweden	1272 (86.9%)	6220 (85.1%)	961 (96.8%)	1935 (96.7%)	982 (86.5%)	1024 (83.9%)
Other Nordic countries	60 (4.1%)	284 (3.9%)	17 (1.7%)	31 (1.6%)	46 (4.1%)	52 (4.3%)
EU28	72 (4.9%)	391 (5.3%)	13 (1.3%)	26 (1.3%)	57 (5.0%)	70 (5.7%)
Non-EU	59 (4.1%)	416 (5.7%)	2 (0.2%)	10 (0.5%)	50 (4.4%)	74 (6.1%)
**Age at death, mean (SD) [N]**	70.5 (9.8) [1024]		69.2 (9.3) [685]		70.8 (9.9) [803]	
**BMI at diagnosis, mean (SD) [N]**	23.9 (4.2) [1027]		23.8 (4.0) [676]		24 (4.2) [799]	
**ALSFRS-R at diagnosis, mean (SD) [N]**	36.4 (8.2) [902]		37.1 (8.0) [603]		36.7 (8.1) [691]	
**Progression rate at diagnosis, median (p25- p75) [N]**	0.6 (0.3–1.1) [856]		0.6 (0.3–1.0) [571]		0.6 (0.3–1.1) [654]	
**Gastrostomy, N (%)**						
PEG	287 (19.6%)		188 (18.9%)		218 (19.2%)	
RIG	10 (0.7%)		6 (0.6%)		8 (0.7%)	
Wizelfistel	1 (0.1%)		1 (0.1%)		1 (0.1%)	
No	1165 (79.7%)		798 (80.4%)		908 (80%)	
**Invasive ventilation, N (%)**						
Yes	20 (1.4%)		15 (1.5%)		15 (1.3%)	
No	1443 (98.6%)		978 (98.5%)		1119 (98.7%)	
**Diagnostic delay in months, median (p25- p75) [N]**	12.4 (7.6–19.8) [1315]		12.8 (7.7–20.1) [890]		12.3 (7.4–19.7) [1017]	
**Dementia, N (%)**						
Yes	82 (5.6%)		40 (4.0%)		69 (6.2%)	
No	451 (30.8%)		297 (29.9%)		339 (29.8%)	
Missing	930 (63.6%)		656 (66.1%)		727 (64%)	
**Familial ALS, N (%)**						
Sporadic	497 (33.9%)		316 (31.8%)		372 (32.7%)	
Familial	60 (4.1%)		44 (4.4%)		46 (4.1%)	
Missing	906 (62.0%)		633 (63.8%)		717 (63.2%)	
**Onset site, N (%)**						
Bulbar	276 (18.9%)		169 (17%)		207 (18.2%)	
Spinal	468 (32.0%)		306 (30.8%)		357 (31.5%)	
Other	63 (4.3%)		44 (4.4%)		49 (4.4%)	
Missing	656 (44.8%)		474 (47.8%)		522 (45.9%)	

BMI: body mass index (Kg/m^2^), EU28: European countries excluding the Nordic countries, MND: Motor Neuron Disease, Non-EU: Non-European countries, PEG: percutaneous endoscopic gastrostomy, p25: 25th percentile, p75: 75th percentile, RIG: radiologically inserted gastrostomy, SES: socioeconomic status; SD: standard deviation.

Diagnosis of any CVD, thromboembolic disease, hypertension, or cerebrovascular disease, within 1 year before the index date was associated with higher odds of MND (OR ranges from 2.36 to 3.71; Supplementary Table S3). However, a diagnosis of diabetes mellitus type 2 (OR 0.75; 95% CI 0.59, 0.94) and hypercholesterolemia (OR 0.78; 95% CI 0.67, 0.91) >5 years before the index date was associated with lower odds of MND. After multivariable adjustment, we found in the population comparison that diabetes mellitus type 2 (OR 0.75; 95% CI 0.62, 0.93) and hypercholesterolemia (OR 0.82; 95% CI 0.71, 0.94) >1 year before diagnosis were associated with lower odds for MND, whereas no association was noted for the other CMDs (Table 2). Similar associations were noted when comparing MND patients to their sibling or spouse controls, apart from hypercholesterolemia for which the association was attenuated in sibling comparison. The inverse association of diabetes was only noted in males, whereas the inverse association of hypercholesterolemia was more prominent in females than males (Supplementary Table S4). Any CVD, hypertension, diabetes, and hypercholesterolemia >1 year before the index date was associated with lower odds for MND among individuals over 67 years (i.e., mean age of MND diagnosis), whereas a history of any CVD >1 year before the index date was also associated with higher odds of MND among individuals below 67 years.

**Table 2:** Adjusted odds ratio (OR) with 95% confidence interval (CI) of MND in relation to any cardiovascular disease, arrythmia, heart failure, thromboembolic disease, hypertension, cerebrovascular disease, ischemic heart disease, diabetes, and hypercholesterolemia more than one year before MND diagnosis, using a conditional logistic regression model^a^.

Diseases	No. of cases/population controls	OR (95% CI)	No. of cases/sibling controls	OR (95% CI)	No. of cases/spouse controls	OR (95% CI)
Any cardiovascular disease	725/2809	0.98 (0.84–1.15)	433/709	1.08 (0.85–1.37)	465/469	1.09 (0.85–1.40)
Arrhythmia	143/565	1.01 (0.83–1.24)	85/161	0.77 (0.56–1.06)	99/122	**0.68 (0.50–0.93)**
Heart failure	39/190	0.84 (0.59–1.20)	23/52	0.71 (0.41–1.21)	27/31	0.63 (0.36–1.11)
Thromboembolic disease	17/87	0.83 (0.49–1.41)	8/28	0.51 (0.23–1.15)	11/19	0.55 (0.25–1.22)
Hypertension	645/2590	0.91 (0.79–1.05)	385/628	1.09 (0.87–1.37)	414/427	1.00 (0.79–1.26)
Cerebrovascular disease	65/303	0.88 (0.67–1.17)	35/84	0.70 (0.45–1.10)	47/57	0.69 (0.45–1.06)
Ischemic heart disease	119/576	0.85 (0.69–1.05)	69/118	1.01 (0.72–1.42)	83/89	0.76 (0.54–1.06)
Diabetes mellitus type 2	126/693	**0.75 (0.62–0.93)**	75/154	0.73 (0.53–1.01)	79/102	**0.67 (0.47–0.94)**
Hypercholesterolemia	407/1845	**0.82 (0.71–0.94)**	238/399	1.02 (0.82–1.28)	273/281	0.90 (0.72–1.13)

Estimates in bold indicate statistical significance.

a Adjusted for age at diagnosis, sex, socioeconomic status, educational attainment, and country of birth.

MND patients were followed for a median (IQR) of 1.34 (0.64 to 2.37) years after diagnosis. During this time, we observed 1024 deaths or initiation of invasive ventilation. MND patients with a history of any CVD (HR 1.43; 95% CI 1.13, 1.81), arrhythmia (HR 1.42; 95% CI 1.04, 1.93), heart failure (HR 1.79; 95% CI 1.02, 3.14), hypertension (HR 1.41; 95% CI 1.12, 1.77), and hypercholesterolemia (HR 1.28; 95% CI 1.01, 1.62) before diagnosis had a higher mortality compared to patients without such, after accounting for the longitudinal changes of ALSFRS-R (Table 3). The associations for any CVD, hypertension, and diabetes mellitus type 2 were stronger in females than males whereas the association for cerebrovascular disease was stronger among patients aged 67 years or older at diagnosis (Supplementary Table S5). Similar estimates were observed when we used time since onset as the time scale (Supplementary Table S6). Findings were also similar when using Cox model after accounting for the ALSFRS-R at diagnosis (Supplementary Table S7).

**Table 3:** Adjusted hazard ratio (HR) with 95% confidence interval (CI) for the risk of death after MND diagnosis in relation to a history of any cardiovascular disease, arrythmia, heart failure, thromboembolic disease, hypertension, cerebrovascular disease, ischemic heart disease, diabetes, and hypercholesterolemia before diagnosis, after taking into account the longitudinal changes of ALSFRS-R in the time-to-event analysis using a joint longitudinal and survival model.

Diseases	Number of events (IR)^a^	HR (95% CI)^b^
Any cardiovascular disease	639 (51.88)	**1.43 (1.13–1.81)**
Arrhythmia	134 (62.82)	**1.42 (1.04–1.93)**
Heart failure	46 (77.72)	**1.79 (1.02–3.14)**
Thromboembolic disease	20 (51.77)	1.52 (0.78–2.94)
Hypertension	572 (53.48)	**1.41 (1.12–1.77)**
Cerebrovascular disease	80 (56.15)	1.45 (0.94–2.24)
Ischemic heart disease	112 (66.00)	1.12 (0.77–1.62)
Diabetes mellitus type 2	103 (57.29)	1.51 (0.99–2.30)
Hypercholesterolemia	358 (53.66)	**1.28 (1.01**–**1.62)**

Estimates in bold indicate statistical significance.

a IR: incidence rate of death or use of invasive ventilation, per 100 person-years.

b Adjusted for age at diagnosis, sex, onset site, diagnostic delay, body mass index at diagnosis, progression rate at diagnosis, and the time-varying ALSFRS-R.

After adjusting for age at diagnosis, sex, onset site, diagnostic delay, and BMI at diagnosis, we found that MND patients with a history of heart failure (β −6.14; 95% CI −10.8, −1.48), hypertension (β −2.79; 95% CI −4.71, −0.87), ischemic heart disease (β −6.51; 95% CI −9.29, −3.74), and hypercholesterolemia (β −3.15; 95% CI −5.09, −1.22) before diagnosis experienced faster functional decline, compared to other patients (Supplementary Table S8). Similar results were noted after further adjustment for ALSFRS-R at diagnosis. MND patients with a pre-diagnostic CMD tended to be older and had lower ALSFRS-R and higher BMI at the time of diagnosis (Supplementary Table S9). Estimates were largely similar when we excluded individuals with a diagnosis of non-ALS MND (Supplementary Tables S10 and S11).

The cluster analysis identified two groups of patients with MND (Table 4 and Supplementary Table S12). Compared to patients in Cluster 1, patients in Cluster 2 tended to be diagnosed later, had worse functional status and higher BMI at diagnosis, lower educational attainment and socioeconomic status, and a higher prevalence of pre-diagnostic CMDs (Table 5). Patients in Cluster 2 also had a higher mortality (HR 1.45; 95% CI 1.14, 1.84, after accounting for the longitudinal changes of ALSFRS-R) and a faster functional decline of ALSFRS-R score over time (β −2.92; 95% CI −4.81, −1.02).

**Table 4:** Adjusted hazard ratio (HR) with 95% confidence interval (CI) for the risk of death after MND diagnosis in relation to belonging to different patient clusters.

Clusters	Number of events (IR)^a^	HR (95% CI)^b^	HR (95% CI)^c^
Cluster 1	271 (34.42)	Ref	Ref
Cluster 2	134 (49.04)	1.33 (1.06, 1.68)	1.45 (1.14, 1.84)

a IR: Incidence rate per 100 person-years.

b Adjusted for socioeconomic status, educational attainment, and country of birth.

c Adjusted for the time-varying ALSFRS-R, socioeconomic status, educational attainment, and country of birth.

**Table 5:** Clinical and sociodemographic characteristics of the patient clusters identified among MND patients.

Characteristics	Cluster 1			Cluster 2			p-value
**Number of participants, N**	422			193			
**Males, N (%)**	176 (41.7%)			153 (79.3%)			<0.001
**Age at diagnosis, median (p25–p75)**	67 (57–73)			72 (65–77)			<0.001
**Age at death, median (p25–p75)**	70 (63–76)			73 (67–77)			<0.001
**BMI at diagnosis, mean (SD)**	23.7 (4.1)			24.6 (4.1)			0.02
**ALSFRS-R at diagnosis, mean (SD)**	37.7 (7.9)			35.8 (8.0)			0.005
**Gastrostomy, N (%)**	113 (26.8%)			52 (26.9%)			0.9
**Invasive ventilation, N (%)**	15 (3.6%)			3 (1.6%)			0.2
**Diagnostic delay in months, median (p25–p75)**	12.5 (7.5, 19.5)			12.2 (8.2, 20)			0.6
**Dementia, N (%)**	39 (9.2%)			29 (15.0%)			0.06
**Familial ALS, N (%)**	39 (9.2%)			5 (2.6%)			0.004
**Onset site, N (%)**	Bulbar	Spinal	Other	Bulbar	Spinal	Other	
	146 (34.6%)	252 (59.7%)	24 (5.7%)	63 (32.6%)	110 (57.0%)	20 (10.4%)	0.1
**SES, N (%)**							
No educational requirements	10 (2.4%)			8 (4.1%)			0.03
Highschool degree required	146 (34.6%)			78 (40.4%)			
University studies required ≤3	84 (19.9%)			34 (17.6%)			
University studies required >3	153 (36.3%)			45 (23.3%)			
Missing	29 (6.9%)			28 (14.5%)			
**Educational attainment, N (%)**							
<9 years	20 (4.7%)			23 (11.9%)			<0.001
9–10 years	37 (8.8%)			18 (9.3%)			
Upper secondary	149 (35.3%)			84 (43.5%)			
Post-secondary <2 years	28 (6.6%)			11 (5.7%)			
Post-secondary ≥2 years	179 (42.4%)			55 (28.5%)			
Postgraduate	6 (1.4%)			0 (0%)			
Missing	3 (0.7%)			2 (1.0%)			
**Country of birth, N (%)**							
Sweden	354 (83.9%)			163 (84.5%)			0.04
Other Nordic countries	25 (5.9%)			6 (3.1%)			
EU28	18 (4.3%)			17 (8.8%)			
Non-EU	25 (5.9%)			7 (3.6%)			
**Cardiometabolic disease, N (%)**							
Arrhythmia	40 (9.5%)			40 (20.7%)			<0.001
Heart failure	8 (1.9%)			13 (6.7%)			0.002
Thromboembolic disease	6 (1.4%)			7 (3.6%)			0.08
Hypertension	108 (25.6%)			174 (90.2%)			<0.001
Cerebrovascular disease	18 (4.3%)			24 (12.4%)			<0.001
Ischemic heart disease	1 (0.2%)			54 (28.0%)			<0.001
Diabetes mellitus type 2	3 (0.7%)			51 (26.4%)			<0.001
Hypercholesterolemia	65 (15.4%)			127 (65.8%)			<0.001

BMI: body mass index (Kg/m2), EU28: European countries excluding the Nordic countries, Non-EU: Non-European countries, p25: 25th percentile, p75: 75th percentile, SES: socioeconomic status, SD: standard deviation.

## Discussion

This nationwide register-based study examined the link between CMDs and the risk and progression of MND. History of diabetes mellitus type 2 or hypercholesterolemia was associated with a lower risk for future MND, whereas MND patients with a history of any CVD, cardiac arrhythmia, heart failure, hypertension, and hypercholesterolemia before diagnosis had an increased mortality and faster functional decline after diagnosis. Cluster analysis identified two groups of MND patients. Patients segregating into one cluster demonstrated lower ALSFRS-R score, higher age, and higher prevalence of CMDs at the time of diagnosis as well as worse prognostic outcomes, including higher mortality and faster functional decline, compared to patients segregating into the other cluster.

We found a lower risk for MND among individuals with diabetes mellitus type 2, regardless of whether the diabetes was diagnosed >1 or >5 years before MND diagnosis, in agreement with previous studies.^5^, ^6^, ^7^^,^^34^^,^^35^ Given the lack of specific codes for diabetes mellitus type 2 in ICD-9, among the study participants with a diagnosis of diabetes during 1987–1996, some might have had diabetes mellitus type 1. Therefore, the observed association for diabetes type 2 might be biased toward null, as diabetes type 1 has been reported to be associated with a higher risk of ALS.^6^^,^^36^ However, given the small number of such participants (n = 48), this bias is unlikely substantial.

The existing evidence for a link between hypercholesterolemia and risk of MND is less conclusive. A history of hypercholesterolemia was shown to be associated with a lower risk for ALS in one study.^11^ However, other studies showed a positive association^13^ or a null association.^9^ These inconsistent findings might be partially explained by a lack of representativeness of the study sample (e.g., cases are not representative of all ALS patients in the source population whereas controls are not representative of the source population that gives rise to the ALS patients). In the present study, hypercholesterolemia was less prevalent among MND patients than controls more than five years before diagnosis. However, in the year before diagnosis, hypercholesterolemia tended to be more prevalent among MND patients, though not reaching statistical significance. As a result, using different time windows to examine the link between hypercholesterolemia and MND risk might lead to different conclusions. Finally, the link between lipid metabolism and MND is likely more complicated than expected. For instance, previous Mendelian randomization (MR) studies have suggested a causal link between higher lipid levels, including total cholesterol, and higher risk of ALS.^14^^,^^15^ However, the MR studies considered mostly lipid levels of normal range, whereas the present study addressed clinically diagnosed hypercholesterolemia. Further, clinically diagnosed hypercholesterolemia is most likely treated with lipid lowering medications, which may also modulate ALS risk independently.^10^, ^11^, ^12^ In the sibling comparison, the estimate for hypercholesterolemia became null, suggesting that shared genetic and early-life environmental factors between full siblings may partly explain the association of hypercholesterolemia with MND risk, particularly for hypercholesterolemia over five years before MND diagnosis. However, the contrasting result pattern noted between the population and sibling comparisons needs to be validated in studies of independent samples.

CMDs are linked to chronic low-grade inflammation,^37^ while neuroinflammation has been implicated in the pathogenesis of ALS^38^ and other neurodegenerative disorders.^39^ Elevated levels of proinflammatory cytokines are observed in both CMDs and ALS indicating potentially a shared inflammatory pathway.^39^, ^40^, ^41^ Activated microglia contribute to motor neuron degeneration, while CMDs through systemic inflammation might trigger or exacerbate microglial activation, promoting neuronal damage.^42^^,^^43^ Another potential pathway is oxidative stress which, through mitochondrial dysregulation, has a key role in endothelial dysfunction, insulin resistance and hypertension.^44^ Oxidative stress is also responsible for metabolic stress in muscle and nerve cells, importantly contributing to neurodegeneration.^45^^,^^46^ Finally, endothelial dysfunction due to CMDs such as hypertension and diabetes might result in functional and structural changes in blood vessels of the central nervous system which might subsequently influence neurodegenerative processes.^47^^,^^48^

In the present study, no association was observed between other CMDs and subsequent risk of MND, when examining CMDs diagnosed >1 year before MND diagnosis. This aligns with previous studies on hypertension, cardiac arrhythmia, heart failure,^4^^,^^49^ ischemic heart disease^12^^,^^18^^,^^50^ and cerebrovascular disease.^18^ One study found a higher prevalence of ischemic heart disease among patients with ALS, compared to the general population; however, this study did not adjust for sex or socioeconomic factors, making confounding bias a concern.^51^ Another study used data on Medicare beneficiaries and showed that heart failure, atrial fibrillation, and hypertension were inversely related to the risk of ALS; however, the participants of this study were approximately 10 years older than the participants of the present study.^17^ Our study found that, among individuals over 67 years, a history of hypertension >1 year before diagnosis was associated with a lower risk of MND. Similarly, a history of any CVD was associated with a lower risk for MND among individuals at 67 or above but a higher risk of MND among individuals younger than 67, in the present study. Collectively, these age-specific results suggest a need of taking into account age in disentangling the role of CMDs in MND risk.

Previous studies have found little association between diabetes and MND survival^19^, ^20^, ^21^, ^22^ whereas our study suggested an association between diabetes type 2 and increased mortality after MND diagnosis among females. The lack of results in previous studies might be explained by different reasons, including insufficient statistical power, varying adjustment for other prognostic indicators (e.g., sex),^22^ or restriction of study population to male.^19^ Our null findings on the link between hypertension, heart failure, and cardiac arrhythmias with MND survival were, on the other hand, consistent with previous studies.^4^^,^^52^ Our finding of a positive association between pre-diagnostic hypercholesterolemia and increased mortality after MND diagnosis is also in agreement with previous studies.^9^^,^^23^^,^^24^ In contrast to the relatively uniform findings on dyslipidemia, a higher level of circulating lipids has been shown to be associated with a better^13^^,^^25^ or worse^26^ survival after ALS diagnosis, however, in different studies. As discussed above for ALS risk, different reasons might underline such discrepancy, including studying lipids within clinically normal range versus clinically diagnosed dyslipidemia, the potential influence of lipid-lowering medications, and different adjustment for other prognostic predictors.^53^

Our finding of worse survival among MND patients with a history of cardiovascular abnormalities is regardless supported by clinical studies. MND patients exhibit abnormal blood pressure values likely linked with hypermetabolism.^54^ Structural defects in cardiac magnetic resonance imaging have been reported among ALS patients and attributed to sympathetic hyperactivity.^55^ Autonomic changes in ALS are likely multifaceted. There is sympathetic predominance and vagal withdrawal in the early stage of ALS, followed by late-stage sympathetic denervation.^56^, ^57^, ^58^, ^59^ Reduced neurons in the intermediolateral nucleus correlate with increased cardiac QTc dispersion, a marker of increased sympathetic activity.^58^ Stress-induced cardiomyopathy in ALS patients includes arrhythmias, electrocardiographic changes, and sudden cardiac death, which is a relatively common cause of death among patients with ALS.^55^^,^^60^ Finally, a common electrocardiographic pattern among ALS patients is a “pseudo-infarct” because of the effects of the disease on neuromuscular and autonomic balance.

MND patients of the present study segregated into two clusters with distinct progression and survival profiles. The higher mortality risk and the faster functional decline over time among individuals in Cluster 2 compared to those in Cluster 1 are likely mainly explained by their older age and the greater prevalence of pre-diagnostic CMDs at the time of diagnosis. Cluster 1 is characterized by a higher constellation of familial ALS cases, and as expected, they developed MND earlier and presented with higher functional score at the time of diagnosis. Although MND was the most common cause of death listed for all patients of the study, patients in cluster 2 were more likely to have acute respiratory failure, cardiac arrest, primary hypertension, chronic obstructive pulmonary disease and chronic ischemic heart disease as the underlying or contributory cause of death (n = 53, 35.8%), compared to patients of the cluster 1 (n = 41, 14.8%).

Our study has several strengths. First, it carefully accounted for familial confounding through the use of both population and relative controls. Second, we comprehensively examined the role of CMDs not only on the risk, but also the prognosis, of MND, demonstrating differential roles of CMDs during different stages of MND development. The fact that we could study CMDs at different time windows before MND diagnosis provides a unique opportunity to potentially differentiate upstream and downstream CMDs events, relative to onset of MND. Specifically, we showed that MND patients had a higher prevalence of CMDs during the year before diagnosis, but they had similar prevalence of CMDs >1 year before diagnosis and a lower prevalence of diabetes mellitus type 2 and hypercholesterolemia >1 year (also >5 years) before diagnosis, compared to controls. Further, we are the first to identify clusters of MND patients, incorporating information on clinical characteristics and pre-diagnostic CMDs, which are indicative of patient outcomes (i.e., survival and functional decline after diagnosis). Another strength of this study is the population-based design, the large sample size, the complete follow-up, and rich data on clinical characteristics and sociodemographic factors of the study population.

This study also has some limitations. We identified CMDs through a diagnosis made through specialist (via the Swedish Patient Register) or primary (i.e., prescription of CMD medications) care. Although such approach is both sensitive and specific,^32^ we did not identify CMDs not attended by specialist care or treated with medications. Second, to adjust for residual confounding due to unknown and unmeasured confounders, we used a novel design with multiple control groups, including population, sibling, and spouse controls. Using full siblings as controls allows to control for confounding due to shared genetics (partially) and early-life environmental factors including familial socioeconomic status between siblings. Using spouse controls allows to control for confounding due to shared adult environment including diet, socioeconomic factors, and environmental exposures between spouses. Contrasting results between the use of different control groups helped therefore to shed light on potential familial confounding in population comparison as well as any bias that may arise from using relative controls (e.g., overmatching). However, residual confounding due to factors not fully shared between relatives may still bias the results to some extent. If an unmeasured confounder is a risk factor for MND but a protective factor for hypercholesterolemia or diabetes, the observed association would be an overestimate of the true association. Conversely, if the unmeasured confounder is a risk factor for both MND and hypercholesterolemia/diabetes, such residual confounding would lead the result towards null, underestimating the true association. For example, smoking is positively associated with the risk of both type 2 diabetes mellitus^61^ and MND,^62^ whereas in our study, we found that type 2 diabetes mellitus might be protective against MND. Not accounting for smoking in this analysis might therefore have underestimated this inverse association. Third, the analysis on disease progression was restricted to patients with complete information on ALSFRS-R and site of onset; as a result, these findings are mostly representative of MND patients in the regions where information on both variables is highly complete in the MND Quality Registry (e.g., Stockholm and western part of Sweden). Fourth, the ALSFRS-R scale was used as a measure of disease progression in the present study, including patients with ALS, PSMA, PLS, and unspecified MND. As PLS affects almost purely upper motor neurons and progresses slowly,^63^ the ALSFRS-R scale might not be the most sensitive measure to study disease progression for patients with PLS. Although a specific scale has been developed for PLS, it needs to be administered by trained personnel whereas its effectiveness and correlations with other disease biomarkers await to be evaluated.^64^ As the Gold Coast criteria classify PSMA patients as ALS,^65^ the ALSFRS-R scale is a likely suitable measure for disease progression in patients with PSMA. In the present study, patients with PLS demonstrated better survival as well as slower decline in ALSFRS-R scores over time, compared to other patient groups, whereas the differences were less clear among patients with ALS, PSMA, or unspecified MND (data not shown). Regardless, future studies are needed to assess the usefulness of ALSFRS-R scale for different types of MND. Finally, the cluster analysis is exploratory in nature and needs to be validated in independent samples to examine whether clusters with different clinical characteristics and history of CMDs can be identified in other MND populations.

### Conclusion

While cardio- and cerebro-vascular diseases did not appear to be associated with the risk of MND, diabetes mellitus type 2 and hypercholesterolemia were associated with a lower future risk of MND. The finding of increased incidence of most CMDs during the year before MND diagnosis highlights the burden of clinical MND disease on the cardiometabolic system. Furthermore, most of the CMDs were indicative of a poor prognosis after MND diagnosis, including higher mortality and faster functional decline. Our study represents the most comprehensive investigation to date of the influence of CMDs on the risk and progression of MND, underscoring the importance of closely monitoring the cardiometabolic health of MND patients during the course of the disease.

## Contributors

CC and FF conceptualized the study and were responsible for data curation and validation. CC and FF accessed and verified the underlying data reported in the manuscript. All authors contributed to project administration and provided necessary resources. FF and CI secured funding for the study. CC, FF, AL, and CI were responsible for the investigation methods, while CC, FF, and AL for the statistical methodology. Decisions regarding statistical software were made by CC and AL. FF, CI, CS, and AL supervised the study. CC conducted the analysis and created any visualisations while also drafted the original manuscript. All authors reviewed and edited the manuscript. All authors have read and approved the final version of the manuscript.

## Data sharing statement

The data of this study cannot be shared publicly according to Swedish and European regulations. Please contact the corresponding authors for more information.

## Declaration of interests

Caroline Ingre has consulted for Cytokinetics, Pfizer, BioArctic, Novartis, Tikomed, Ferrer, Amylyx, Prilenia, and Mitsubishi. She is also a board member of Tobii Dynavox; all outside the submitted work.
