# Supplementary material for: Association between cardiometabolic diseases and the risk and progression of motor neuron diseases in Sweden: a population-based case–control study
Source: Lancet Reg Health Eur. 2024 Dec 10;49:101173. doi: 10.1016/j.lanepe.2024.101173 (PMC11697398; doi:10.1016/j.lanepe.2024.101173)

Supplementary Table 1. ICD and ATC codes for the ascertainment of cardiometabolic diseases

| Diseases | ICD10 | ICD9 | ATC |
| --- | --- | --- | --- |
| Ischemic heart disease | I20-I25 | 410-414 |  |
| Cerebrovascular disease | I60-I69 | 430-434, 436-438 |  |
| Thromboembolic disease | I26, I74 | 415, 444-445 |  |
| Hypertension | I10-I15 I67.4 | 401-405, 437C | C02,C03, C07-C09 Exclusion 1) if C03C, C03D, C07A, C09 in combination with an I50 or I42 diagnosis; 2) if C07 or C08D in combination with I47 or I48, I49 diagnosis |
| Heart failure | I25.5, I42, I50 | 428, 429X, 425, 421A |  |
| Cardiac arrhythmia | I44-I49 | 426, 427 | C01B |
| Diabetes mellitus type 2 | E11-E14 | 250 | A10 |
| Hypercholesterolemia | E780- E785 | 272A-E | C10 |
| Any cardiovascular disease | I10–I70 | 400-440 | C02,C03, C07-C09 Exclusion 1) if C03C, C03D, C07A, C09 in combination with an I50 or I42 diagnosis; 2) if C07 or C08D in combination with I47 or I48, I49 diagnosis  C01B |

Supplementary Table 2. Number (%) of patients missing ALSFRS-R and site of onset, and number (%) of patients included in the analysis of disease progression.

| Region of hospital | Number of patients | Number (%) of patients missing ALSFRS-R | Number (%) of patients missing site of onset | Number (%) of patients with both ALSFRS-R and site of onset (i.e., included in analyses of disease progression) |
| --- | --- | --- | --- | --- |
| North | 93 | 61 (65.59%) | 80 (86.02%) | 7 (7.52%) |
| Stockholm | 624 | 93 (14.90%) | 57 (9.13%) | 498 (79.81%) |
| South Eastern | 124 | 88 (70.97%) | 115 (92.74%) | 5 (4.03%) |
| South | 178 | 100 (56.18%) | 147 (82.58%) | 21 (11.79%) |
| Uppsala-Örebro | 198 | 166 (83.83%) | 184 (92.93%) | 3 (1.51%) |
| West | 246 | 53 (21.54%) | 73 (29.67%) | 153 (62.19%) |

Supplementary Table 3. Adjusted odds ratio (OR) with 95% confidence interval (CI) of MND in relation to any cardiovascular disease, arrythmia, heart failure, thromboembolic disease, hypertension, cerebrovascular disease, ischemic heart disease, diabetes, and hypercholesterolemia at 0-1, 1-5, or >5 years before MND diagnosis, using a conditional logistic regression model- analysis comparing MND patients with population controls**^*^**

| Diseases | 0-1 year | 1-5 years | >5 years |
| --- | --- | --- | --- |
| Any cardiovascular disease | **2.94 (1.99- 4.32)** | 1.02 (0.78- 1.33) | 0.97 (0.82- 1.16) |
| Arrhythmia | 1.15 (0.70- 1.87) | 1.03 (0.73- 1.44) | 1.02 (0.79- 1.30) |
| Heart failure | 1.45 (0.81- 2.59) | 0.78 (0.46- 1.32) | 0.92 (0.58- 1.47) |
| Thromboembolic disease | **3.71 (1.79- 7.69)** | 1.02 (0.48- 2.16) | 0.72 (0.36- 1.47) |
| Hypertension | **2.36 (1.64- 3.40)** | 1.16 (0.91- 1.47) | 0.86 (0.74- 1.01) |
| Cerebrovascular disease | **3.57 (2.14- 5.97)** | 0.89 (0.54- 1.46) | 0.89 (0.63- 1.24) |
| Ischemic heart disease | 1.18 (0.59- 2.34) | 0.79 (0.52- 1.22) | 0.86 (0.67- 1.09) |
| Diabetes mellitus type 2 | 1.14 (0.64- 2.03) | 0.79 (0.54- 1.17) | **0.75 (0.59- 0.94)** |
| Hypercholesterolemia | 1.42 (0.97- 2.08) | 0.98 (0.75- 1.26) | **0.78 (0.67- 0.91)** |
| **^*^**Adjusted for age at diagnosis, sex, socioeconomic status, educational attainment, and country of birth. | | | |

Supplementary Table 4. Adjusted odds ratio (OR) with 95% confidence interval (CI) of MND in relation to any cardiovascular disease, arrythmia, heart failure, thromboembolic disease, hypertension, cerebrovascular disease, ischemic heart disease, diabetes, and hypercholesterolemia more than one year before MND diagnosis, using a conditional logistic regression model - stratified analysis by sex and age**^*^**

| Diseases | Male | Female | ≥67 years | <67 years |
| --- | --- | --- | --- | --- |
| Any cardiovascular disease | 1.10 (0.89- 1.36) | 0.87 (0.69- 1.09) | **0.71 (0.58- 0.87)** | **1.47 (1.16- 1.85)** |
| Arrhythmia | 1.19 (0.93- 1.53) | 0.76 (0.52- 1.09) | 0.99 (0.79- 1.24) | 1.14 (0.73- 1.78) |
| Heart failure | 1.00 (0.66- 1.51) | 0.53 (0.25- 1.12) | 0.77 (0.51- 1.15) | 1.22 (0.56- 2.70) |
| Thromboembolic disease | 0.82 (0.43- 1.58) | 0.83 (0.34- 2.03) | 0.58 (0.30- 1.14) | 2.30 (0.92- 5.74) |
| Hypertension | 0.99 (0.81- 1.20) | 0.83 (0.67- 1.02) | **0.74 (0.61- 0.89)** | 1.23 (0.99- 1.54) |
| Cerebrovascular disease | 0.93 (0.65- 1.33) | 0.82 (0.52- 1.29) | 0.87 (0.64- 1.19) | 0.90 (0.44- 1.86) |
| Ischemic heart disease | 0.91 (0.71- 1.18) | 0.73 (0.49- 1.08) | 0.84 (0.66- 1.06) | 0.93 (0.56- 1.54) |
| Diabetes mellitus type 2 | **0.64 (0.49- 0.83)** | 1.01 (0.73- 1.39) | **0.74 (0.58- 0.94)** | 0.83 (0.56- 1.22) |
| Hypercholesterolemia | 0.84 (0.70- 1.02) | **0.78 (0.63- 0.96)** | **0.77 (0.65- 0.92)** | 0.94 (0.73- 1.21) |
| **^*^**Adjusted for age at diagnosis, sex, socioeconomic status, educational attainment, and country of birth. | | | | |

Supplementary Table 5. Adjusted hazard ratio (HR) with 95% confidence interval (CI) for the risk of death after MND diagnosis in relation to a history of any cardiovascular disease, arrythmia, heart failure, thromboembolic disease, hypertension, cerebrovascular disease, ischemic heart disease, diabetes, and hypercholesterolemia before diagnosis, after taking into account the longitudinal changes of ALSFRS-R in the time-to-event analysis using a joint longitudinal and survival model– stratified analysis by sex, onset site and age at diagnosis^*^

|  | Interaction with sex | | | Interaction with onset site | | | Interaction with age at diagnosis | | | |
| --- | --- | --- | --- | --- | --- | --- | --- | --- | --- | --- |
| Diseases | Males | Females | p | Spinal | Non- spinal | p | ≥67 years | <67 years | p |  |
| Any cardiovascular disease | 1.14 (0.83- 1.55) | **1.83 (1.32- 2.54)** | **0.03** | 1.44 (1.07- 1.95) | 1.40 (0.99- 1.98) | 0.9 | 1.40 (1.04- 1.88) | 1.53 (1.07- 2.19) | 0.7 |  |
| Arrhythmia | 1.37 (0.92- 2.04) | 1.49 (0.93- 2.38) | 0.7 | 1.53 (1.04- 2.26) | 1.20 (0.74- 1.94) | 0.4 | 1.47 (1.04- 2.07) | 1.39 (0.70- 2.78) | 0.9 |  |
| Heart Failure | 1.66 (0.85- 3.24) | 2.14 (0.84- 5.45) | 0.7 | 2.45 (1.11- 5.40) | 1.38 (0.65- 2.92) | 0.3 | 1.84 (1.03- 3.28) | 2.69 (0.37- 19.6) | 0.7 |  |
| Thromboembolic disease | 1.25 (0.57- 2.76) | 2.75 (0.84- 8.98) | 0.3 | 1.24 (0.49- 3.11) | 2.07 (0.82- 5.18) | 0.4 | 1.43 (0.66- 3.11) | 2.50 (0.77- 8.15) | 0.4 |  |
| Hypertension | 1.04 (0.77- 1.42) | **1.99 (1.43- 2.76)** | **<0.01** | 1.32 (0.98- 1.79) | 1.54 (1.11- 2.15) | 0.5 | 1.29 (0.98- 1.70) | 1.74 (1.20- 2.52) | 0.2 |  |
| Cerebrovascular disease | 1.25 (0.70- 2.23) | 1.81 (0.92- 3.52) | 0.4 | 1.58 (0.80- 3.11) | 1.26 (0.72- 2.20) | 0.6 | **1.81 (1.16- 2.82)** | 0.22 (0.03- 1.72) | **0.05** |  |
| Ischemic heart disease | 1.16 (0.75- 1.80) | 1.04 (0.54- 1.98) | 0.8 | 1.44 (0.89- 2.33) | 0.87 (0.51- 1.49) | 0.2 | 1.13 (0.76- 1.69) | 1.56 (0.62- 3.91) | 0.5 |  |
| Diabetes mellitus type 2 | 1.01 (0.58- 1.78) | **3.15 (1.71- 5.80)** | **0.01** | 1.67 (0.95- 2.93) | 1.29 (0.71- 2.36) | 0.5 | 1.42 (0.86- 2.34) | 2.06 (0.98- 4.29) | 0.4 |  |
| Hypercholesterolemia | 1.16 (0.85- 1.59) | 1.44 (1.02- 2.04) | 0.4 | 1.20 (0.87- 1.66) | 1.42 (1.01- 1.99) | 0.5 | 1.24 (0.94- 1.63) | 1.51 (0.98- 2.32) | 0.5 |  |
| P-for-interaction  ^*^Adjusted for age at diagnosis, sex, onset site, diagnostic delay, body mass index at diagnosis, progression rate at diagnosis, and the time-varying ALSFRS-R. | | | | | | | | | |  |

Supplementary Table 6. Adjusted hazard ratio (HR) with 95% confidence interval (CI) for the risk of death after MND onset in relation to a history of any cardiovascular disease, arrythmia, heart failure, thromboembolic disease, hypertension, cerebrovascular disease, ischemic heart disease, diabetes, and hypercholesterolemia before onset, after taking into account the longitudinal changes of ALSFRS-R in the time-to-event analysis using a joint longitudinal and survival model.

| Diseases | Number of events (IR)^*^ | HR (95% CI)^†^ |
| --- | --- | --- |
| Any cardiovascular disease | 642 (26.98) | **1.50 (1.19- 1.90)** |
| Arrhythmia | 134 (29.44) | **1.50 (1.10- 2.04)** |
| Heart failure | 46 (35.11) | 1.54 (0.89- 2.66) |
| Thromboembolic disease | 20 (22.43) | 1.18 (0.62- 2.25) |
| Hypertension | 575 (27.71) | **1.43 (1.14- 1.80)** |
| Cerebrovascular disease | 80 (27.28) | 1.26 (0.82- 1.94) |
| Ischemic heart disease | 112 (34.06) | 1.12 (0.78- 1.61) |
| Diabetes mellitus type 2 | 104 (27.91) | 1.48 (0.98- 2.22) |
| Hypercholesterolemia | 359 (27.25) | **1.30 (1.03- 1.65)** |
| ^*^IR: incidence rate of death or use of invasive ventilation, per 100 person-years.  ^†^Model adjusted for age at diagnosis, sex, onset site, diagnostic delay, body mass index at diagnosis, progression rate at diagnosis, and the time-varying ALSFRS-R. | | |

Supplementary Table 7. Adjusted hazard ratio (HR) with 95% confidence interval (CI) for the risk of death after MND diagnosis in relation to a history of any cardiovascular disease, arrythmia, heart failure, thromboembolic disease, hypertension, cerebrovascular disease, ischemic heart disease, diabetes, and hypercholesterolemia before diagnosis, using Cox model

| Diseases | Number of events (IR)^*^ | HR (95% CI)^†^ |
| --- | --- | --- |
| Any cardiovascular disease | 639 (51.88) | **1.41 (1.12- 1.77)** |
| Arrhythmia | 134 (62.82) | **1.38 (1.01- 1.87)** |
| Heart failure | 46 (77.72) | **1.77 (1.01- 3.09)** |
| Thromboembolic disease | 20 (51.77) | 1.41 (0.73- 2.74) |
| Hypertension | 572 (53.48) | **1.38 (1.10- 1.72)** |
| Cerebrovascular disease | 80 (56.15) | 1.40 (0.91- 2.18) |
| Ischemic heart disease | 112 (66.00) | 1.10 (0.76- 1.59) |
| Diabetes mellitus type 2 | 103 (57.29) | 1.43 (0.94- 2.20) |
| Hypercholesterolemia | 358 (53.66) | 1.23 (0.97- 1.55) |
| ^*^IR: incidence rate of death or use of invasive ventilation, per 100 person-years.  ^†^Adjusted for age at diagnosis, sex, onset site, diagnostic delay, body mass index at diagnosis, progression rate at diagnosis, and ALSFRS-R at diagnosis. | | |

Supplementary Table 8. Average change in ALSFRS-R score over time (with a time unit of every 6 months) after MND diagnosis in relation to a history of any cardiovascular disease, arrythmia, heart failure, thromboembolic disease, hypertension, cerebrovascular disease, ischemic heart disease, diabetes, and hypercholesterolemia before diagnosis, using linear mixed models with random intercept and slope

| Diseases | β coefficient (95% CI)^*^ | β coefficient (95% CI)^†^ |
| --- | --- | --- |
| Any cardiovascular disease | -1.56 (-3.62, 0.50) | -1.36 (-3.33, 0.61) |
| Arrhythmia | -0.17 (-2.68, 2.33) | -0.75 (-2.97, 1.48) |
| Heart failure | **-6.14 (-10.8, -1.48)** | **-6.46 (-10.3, -2.58)** |
| Thromboembolic disease | 0.10 (-5.11, 5.32) | 0.63 (-4.03, 5.30) |
| Hypertension | **-2.79 (-4.71, -0.87)** | **-2.49 (-4.29. -0.69)** |
| Cerebrovascular disease | -0.11 (-4.53, 4.31) | 0.58 (-3.79, 4.94) |
| Ischemic heart disease | **-6.51 (-9.29, -3.74)** | **-6.11 (-8.63, -3.60)** |
| Diabetes mellitus type 2 | -2.53 (-5.41, 0.36) | **-2.97 (-5.59, -0.35)** |
| Hypercholesterolemia | **-3.15 (-5.09, -1.22)** | **-2.96 (-4.76, -1.16)** |
| *Adjusted for age at diagnosis, sex, onset site, diagnostic delay, and body mass index at diagnosis.  ^†^Additionally adjusted for ALSFRS-R at diagnosis. | | |

Supplementary Table 9. Clinical characteristics of MND patients with and without a pre-diagnostic cardiometabolic disease

| **Characteristics** | **No** | | | | **Yes** | | | | **P for difference** |
| --- | --- | --- | --- | --- | --- | --- | --- | --- | --- |
| **Number of participants** | 563 | | | | 900 | | | |  |
| **Male, N (%)** | 313 (55.6%) | | | | 501 (55.7%) | | | | 0.9 |
| **Age at diagnosis, mean (SD)** | 62.2 (12.5) | | | | 70.6 (9.8) | | | | <0.001 |
| **Age at death, mean (SD)** | 66.4 (10.3) | | | | 72.7 (8.9) | | | | <0.001 |
| **BMI at diagnosis, mean (SD)** | 23.5 (4.0) | | | | 24.1 (4.3) | | | | 0.01 |
| **ALSFRS-R at diagnosis, mean (SD)** | 37.7 (8.3) | | | | 35.4 (8.1) | | | | <0.001 |
| **Gastrostomy, N (%)** | 115 (20.4%) | | | | 183 (20.3%) | | | | 0.6 |
| **Invasive ventilation, N (%)** | 14 (2.5%) | | | | 6 (0.7%) | | | | 0.004 |
| **Diagnostic delay in months, median (p25- p75)** | 12.4 (7.6, 19.5) | | | | 12.4 (7.5, 20.0) | | | | 0.8 |
| **Dementia, N (%)** | 28 (5%) | | | | 54 (6%) | | | | 0.1 |
| **Familial ALS, N (%)** | Sporadic | | Familial | | Sporadic | | Familial | |  |
|  | 210 (37.2%) | | 35 (6.2%) | | 287 (31.8%) | | 25 (2.8%) | | 0.02 |
| **Onset site** | Bulbar | Spinal | | Other | Bulbar | Spinal | | Other |  |
|  | 95 (16.8%) | 210 (37.2%) | | 22 (3.9%) | 181 (20.0%) | 258 (28.6%) | | 41 (4.7%) | 0.01 |
| BMI: body mass index (Kg/m2), SD: standard deviation | | | | | | | | | |

Supplementary Table 10. Adjusted odds ratio (OR) with 95% confidence interval (CI) of ALS in relation to any cardiovascular disease, arrythmia, heart failure, thromboembolic disease, hypertension, cerebrovascular disease, ischemic heart disease, diabetes, and hypercholesterolemia more than one year before ALS diagnosis, using a conditional logistic regression model- analysis comparing ALS patients with population controls after excluding patients with non-ALS MND diagnoses

| Diseases | No. of cases/ population controls | OR (95%CI)^*^ |
| --- | --- | --- |
| Any cardiovascular disease | 528/ 2002 | 1.03 (0.85- 1.24) |
| Arrhythmia | 109/ 389 | 1.13 (0.89- 1.43) |
| Heart failure | 31/ 129 | 0.99 (0.66- 1.48) |
| Thromboembolic disease | 14/ 64 | 0.94 (0.52- 1.69) |
| Hypertension | 468/ 1871 | 0.90 (0.76- 1.07) |
| Cerebrovascular disease | 46/ 217 | 0.86 (0.61- 1.20) |
| Ischemic heart disease | 84/ 411 | 0.85 (0.66- 1.09) |
| Diabetes mellitus type 2 | 96/ 485 | 0.83 (0.66- 1.05) |
| Hypercholesterolemia | 303/ 1338 | 0.85 (0.72- 1.00) |
| ^*^Adjusted for age at diagnosis, sex, socioeconomic status, educational attainment, and country of birth. | | |

Supplementary Table 11. Adjusted hazard ratio (HR) with 95% confidence interval (CI) for the risk of death after ALS diagnosis in relation to a history of any cardiovascular disease, arrythmia, heart failure, thromboembolic disease, hypertension, cerebrovascular disease, ischemic heart disease, diabetes, and hypercholesterolemia before diagnosis, after taking into account the longitudinal changes of ALSFRS-R in the time-to-event analysis using a joint longitudinal and survival model and excluding patients with non-ALS MND diagnoses

| Diseases | Number of events (IR)^*^ | HR (95% CI)^†^ |
| --- | --- | --- |
| Any cardiovascular disease | 483 (59.96) | **1.42 (1.09- 1.85)** |
| Arrhythmia | 105 (70.55) | 1.23 (0.87- 1.76) |
| Heart failure | 34 (73.39) | 1.57 (0.84- 2.95) |
| Thromboembolic disease | 19 (68.23) | 1.23 (0.63- 2.41) |
| Hypertension | 433 (61.98) | **1.42 (1.10- 1.83)** |
| Cerebrovascular disease | 64 (62.75) | 1.54 (0.97- 2.46) |
| Ischemic heart disease | 83 (70.15) | 1.00 (0.67- 1.49) |
| Diabetes mellitus type 2 | 76 (58.04) | 1.32 (0.84- 2.08) |
| Hypercholesterolemia | 273 (60.25) | 1.21 (0.93- 1.57) |
| ^*^IR: incidence rate of death or use of invasive ventilation, per 100 person-years.  ^†^Model adjusted for age at diagnosis, sex, onset site, diagnostic delay, body mass index at diagnosis, progression rate at diagnosis, and the time-varying ALSFRS-R. | | |

Supplementary Table 12. Average change in ALSFRS-R score over time (with a time unit of every 6 months) after MND diagnosis in relation to belonging to different patient clusters

| **Clusters** | β coefficient (95% CI)^*^ |
| --- | --- |
| **Cluster 1** | Ref |
| **Cluster 2** | -2.92 (-4.81, -1.02) |
| ^*^Adjusted for socioeconomic status, educational attainment, and country of birth. | |

Supplementary Figure 1. Flowchart of the study design including inclusion and exclusion criteria


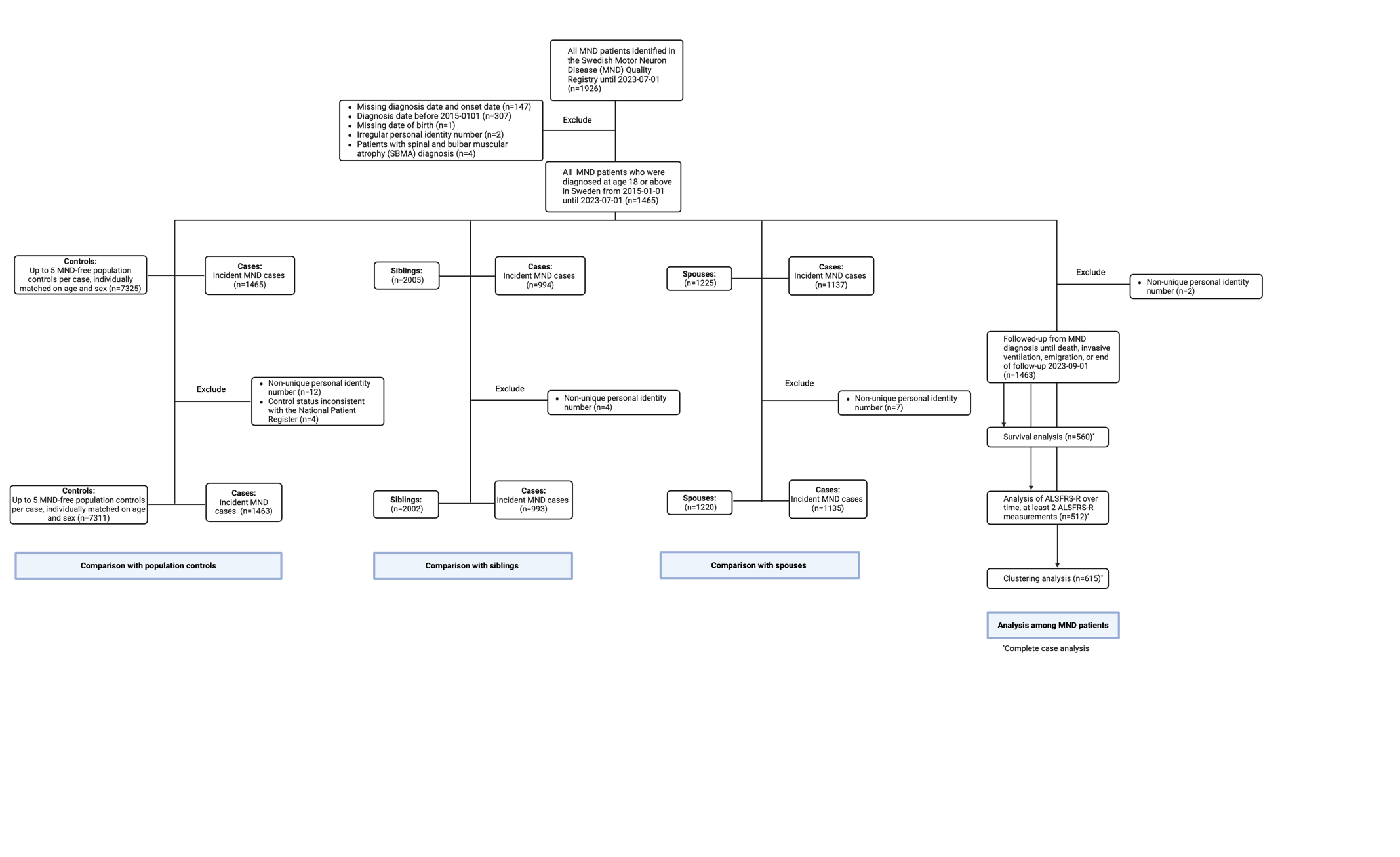

Supplement: Supplementary Figure and Tables [file mmc1.docx]
